# An Update on the Interdisciplinary Dental Care Approach for Geriatric Diabetic Patients

**DOI:** 10.3390/geriatrics8060114

**Published:** 2023-11-25

**Authors:** Zenovia Surlari, Oana Elena Ciurcanu, Dana Gabriela Budala, Oana Butnaru, Ionut Luchian

**Affiliations:** 1 Department of Fixed Prosthodontics, Faculty of Dental Medicine, “Grigore T. Popa” University of Medicine and Pharmacy, 16 Universității Street, 700115 Iasi, Romania; zinovia.surlari@umfiasi.ro; 2 Department of Dental Surgery, Grigore T. Popa University of Medicine and Pharmacy, Universitatii Street 16, 700115 Iasi, Romania; oana.ciurcanu@umfiasi.ro; 3 Department of Implantology, Removable Prostheses, Dental Prostheses Technology, “Grigore T. Popa” University of Medicine and Pharmacy, 16 Universitătii Street, 700115 Iasi, Romania; 4 Department of Biophysics, Faculty of Dental Medicine, “Grigore T. Popa” University of Medicine and Pharmacy, 700115 Iasi, Romania; oana.maria.butnaru@umfiasi.ro; 5 Department of Periodontology, Grigore T. Popa University of Medicine and Pharmacy, Universitatii Street 16, 700115 Iasi, Romania

**Keywords:** diabetes, oral health, saliva, periodontal disease, xerostomia

## Abstract

Diabetes mellitus is a prevalent health issue escalating worldwide that gives rise to numerous problems. Periodontal disorders are recognized as the sixth consequence associated with diabetes mellitus. Research shows that dental health affects overall health, and this knowledge is changing the dental field. The correct choice of glucose goal levels and the optimal selection of glucose-lowering medications are determined by a comprehensive geriatric assessment, an estimate of life expectancy, and a rationale for therapy at regular intervals in elderly diabetics. This article provides an overview of the correlation between diabetes and oral health, with a specific emphasis on xerostomia, periodontal disease, and dental caries. Thus, dentists play a significant role within the allied health profession by contributing to the provision of oral care for those diagnosed with diabetes, with a special focus on geriatric patients.

## 1. Introduction

Diabetes mellitus is one of the world’s most serious public health concerns due to its high and growing prevalence and the various and significant morbidity it causes, harming individuals, health systems, and countries’ economies [1,2]. Recent estimates suggest that 537 million adults worldwide are affected by the disease, with 80% residing in low- and middle-income nations [3].

Furthermore, DM is a multifaceted and insidious condition. It is a medical condition that is distinguished by irregularities in the metabolism of carbohydrates, lipids, and proteins. These abnormalities arise due to either a significant or complete lack of insulin, which is caused by the autoimmune destruction of the pancreatic beta cells responsible for insulin production (known as type 1 diabetes or insulin-dependent diabetes mellitus) [4].

Alternatively, diabetes can also result from the resistance of target tissues to the metabolic effects of insulin, which is commonly associated with obesity (known as type 2 diabetes or non-insulin-dependent diabetes mellitus). Type 1 diabetes accounts for a maximum of 5 percent of primary diabetes cases, while type 2 diabetes encompasses the remaining majority of primary cases [5,6].

Delivery of needed drugs, control of glycemia and other cardiometabolic risk factors, and early screening for problems, all contribute to reduced acute and chronic consequences and increased longevity for those with a diabetes diagnosis [7].

## 2. Defining the Diabetes and Oral Health Connection

The medical community has not yet identified a clear cure for diabetes. The aforementioned condition is well-recognized as the prevailing endocrine ailment, with a substantial impact on approximately 16 million individuals residing within the United States. There is an approximate additional population of 6 million individuals who are affected by diabetes yet remain unaware of their condition. Individuals who lack a thorough diagnosis are exposed to substantial hazards that may lead to the emergence of life-threatening consequences [8,9]. Although some countries have seen a decline in incidence, the prevalence of diabetes has risen in most industrialized and developing nations over the past several decades [10]. There are currently 451.2 million adults with diabetes globally, according to the International Diabetes Federation (IDF), with that number expected to rise to 693.0 million by 2045 if effective preventative strategies are not implemented [11,12].

Over time, diabetes can damage numerous bodily functions and cause life-threatening complications [13]. The complications associated with this condition encompass heightened vulnerability to infections and impaired wound healing. Additionally, individuals may experience neuropathy, retinopathy, and nephropathy, which are classified as microvascular diseases. This condition also leads to accelerated atherosclerosis, resulting in myocardial infarction, coronary artery disease, and strokes.

Macrosomia and other delivery problems are also common among pregnant women with diabetes, as are dental disease and a lowered susceptibility to respiratory viruses. Complications for people with type 1 and type 2 diabetes are similar in nature but might manifest at different rates or in different time frames [14,15,16].

Diabetes mellitus is traditionally divided into autoimmune (T1DM) and non-autoimmune (T2DM) subtypes. Approximately 463 million adults, accounting for 9.3% of the global population, are afflicted with diabetes mellitus (DM), and this number is projected to reach 700 million individuals, constituting 10.9% of the population, by the year 2045 [17]. An extra 374 million individuals are afflicted with prediabetes (preDM), placing them at a heightened risk of acquiring type 2 diabetes mellitus (T2DM) [17].

**Figure 1:**
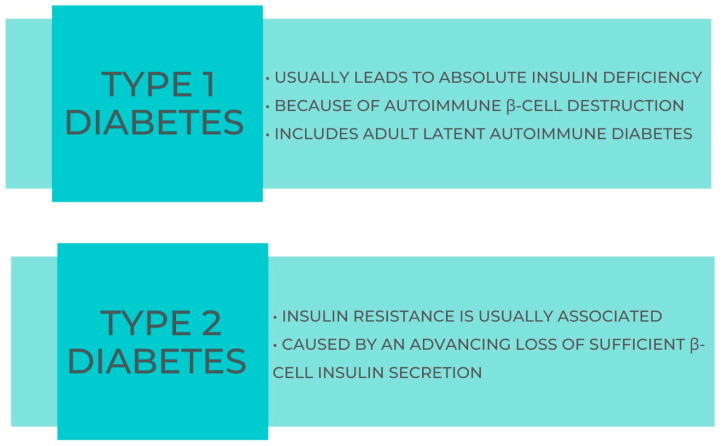
Main characteristics of T1DM and T2DM.

Type 1 diabetes mellitus (T1DM) is characterized by the death of β-cells, resulting in a definitive decrease in insulin output. Type II diabetes mellitus (T2DM), commonly referred to as non-insulin-dependent diabetes mellitus, is the prevailing form of diabetes mellitus. It arises due to a gradual impairment in the production of insulin and/or resistance to the physiological actions of insulin, as shown in Figure 1.

Other forms of diabetes have been identified from a clinico-pathological vantage point; these include monogenic diabetes (also known as juvenile-onset diabetes or neonatal diabetes), diabetes during pregnancy, and possibly a late-onset autoimmune variety (latent autoimmune diabetes in adults). Elevated blood glucose, known as hyperglycemia, is a characteristic feature of diabetes mellitus, along with its associated chronic metabolic problems. The severity of these symptoms tends to be higher in those diagnosed with type 1 diabetes mellitus [18,19,20].

The maintenance of blood glucose levels, also known as glycemic control, is a crucial aspect of the medical treatment of diabetes. The prolonged and severe elevation of blood glucose levels has been linked to the development of both systemic and oral problems [21].

While there is no direct correlation between diabetes and specific oral lesions, extended periods of elevated blood sugar levels can result in many oral symptoms. These may include a burning sensation in the oral mucosa, dry mouth (xerostomia), dental caries, and periodontal diseases such as gingivitis and periodontitis. Ultimately, these oral complications can lead to premature tooth loss [22].

Several studies have demonstrated a higher incidence of dental caries, specifically root caries. However, there is currently no substantiated evidence supporting a direct association. On the contrary, the presence of uncomfortable, movable, and missing teeth [23,24] might contribute to suboptimal nutritional intake, hence heightening the likelihood of developing type 2 diabetes mellitus (T2DM) or experiencing inadequate glucose management in those with pre-existing diabetes [25].

The occurrence of xerostomia in individuals diagnosed with diabetes primarily stems from advanced age and the adverse effects of medication. The connection between diabetes and periodontitis, an irreversible form of periodontal disease marked by the deterioration of the periodontal ligament and alveolar bone, is of great significance.

There is a growing body of research that indicates the reciprocal association between these two diseases. The presence of diabetes elevates the likelihood of developing periodontitis, a condition characterized by inflammation of the gums and the surrounding tissues. Furthermore, the occurrence of periodontal inflammation has a detrimental impact on the regulation of blood sugar levels [26,27].

Existing research has indicated that routine dental appointments have the potential to exert a favorable influence on the management of diabetes and the prevention of its associated complications. This can be achieved through the facilitation of preventive measures, timely identification, and intervention in cases of periodontal disease [28].

The use of rigorous oral hygiene practices has been shown to effectively mitigate oral inflammation and decelerate the progression of periodontal degeneration in individuals diagnosed with diabetes. However, individuals diagnosed with diabetes have demonstrated suboptimal adherence to prescribed oral hygiene practices, including brushing their teeth twice daily, cleaning the spaces between teeth and interdental surfaces at least once a day, and seeking dental care from a professional at least once a year [29,30].

## 3. Oral Complications of Diabetes

In 2007, diabetes afflicted over 250 million individuals globally; by 2030, that number is expected to rise to 350 million [31]. It is projected that by 2050, the world’s senior population will have climbed from its 1980 low of 382 million to 962 million. There are many potential long-term complications associated with both type 1 and type 2 diabetes. Researchers have found that a correlation exists between hyperglycemia and the severity of diabetes complications.

**Figure 2:**
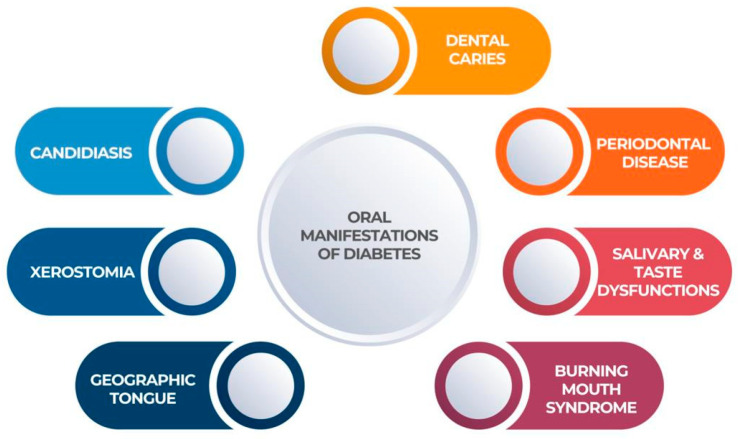
Main complications of DM.

Dry mouth [32], tooth decay [33], periodontal disease and gingivitis [34], oral candidiasis, burning mouth syndrome (BMS) [32], taste disorders, rhino cerebral zygomycosis (mucormycosis), aspergillosis, oral lichen planus [35], geographic tongue [36], fissured tongue [36], delayed wound healing, and an increased incidence of infection are all described as oral manifestations of DM, as can be seen in Figure 2 below:

### 3.1. Salivary Complications

Saliva is a type of exocrine secretion that primarily consists of water, making up approximately 99% of its composition. It also contains a diverse range of electrolytes, including sodium, potassium, calcium, chloride, magnesium, bicarbonate, and phosphate. Additionally, salivary fluid contains various proteins, such as enzymes, immune globulins, antimicrobial factors, mucosal glycoproteins, and small amounts of albumin, as well as polypeptides and oligopeptides [37].

These components play a significant role in maintaining oral health. In addition, glucose and nitrogenous compounds, including urea and ammonia, are also present. The aforementioned components exhibit interdependence and collectively contribute to the diverse physiological roles associated with saliva [38].

The examination of biochemical components in saliva is highly beneficial for diagnosing oral cavity disorders and monitoring overall organism health [30]. According to a substantial body of research, it has been demonstrated that diabetic patients have alterations in the composition of both the organic and inorganic components of their saliva [39].

These changes can be attributed to the presence of autonomic neuropathies, microvascular abnormalities, hormonal imbalances, or a combination of these factors, which are commonly associated with diabetes [39]. When there is a change in the typical conditions of the oral cavity due to a reduction in saliva production or changes in the composition of the saliva, oral health might be compromised, leading to an increased risk of dental caries and tooth decay. Insufficient salivary secretion might lead to the eventual complication of dry, atrophic, and cracking oral mucosa [40].

In addition to the presence of mucositis, ulcers, and desquamation, individuals may also experience an inflamed and depapillated tongue, a frequently observed complication. The recognition and reporting of oral symptoms and consequences in individuals with diabetes mellitus have emerged as a significant complication of this medical condition in recent times.

Xerostomia is commonly reported by people with diabetes, according to several epidemiological studies. Studies have also shown that people with diabetes had lower salivary flow rates than the general population [41].

Particularly in the presence of dehydration and inadequate blood glucose management, these salivary abnormalities may enhance the vulnerability of DM patients to caries and oral infections [42]. The prevalence of diabetes makes it a leading candidate for the title of most common metabolic disease with salivary implications [43].

Impaired nutritional intake can be attributed to significant issues arising from salivary dysfunction, specifically the challenges encountered in lubrication, mastication, taste, and swallowing [44]. A rise in the incidence of dental caries among young individuals with diabetes has been reported, which may be attributed to impaired salivary function [45].

### 3.2. Gingivitis and Periodontal Disease

Recent studies, summarized in a meta-analysis, suggest a positive correlation between glucose disturbances (including diabetes) and periodontal disease [46]. There is also evidence to suggest that periodontal disease raises the likelihood of developing type 2 diabetes [47]. This reciprocal relationship may be explained by the fact that both diseases are fueled by inflammatory processes.

The prevalence of periodontitis is increased two to threefold in patients with diabetes compared to the general population [48], with glycemic control being the most important factor in predicting risk [49], and the severity of periodontitis is increased by a factor of ten in current smokers [50].

In terms of oral complications, diabetes most commonly increases a person’s vulnerability to periodontal disease, which is sometimes referred to as the “sixth complication of diabetes mellitus” [51]. Uncontrolled diabetes increases a patient’s risk of developing periodontal disease. Poor glycemic management causes an individual’s condition to proceed from gingivitis to periodontitis.

Dental infections in diabetic patients have been linked to worsening metabolic regulation; nevertheless, the precise nature of this complex relationship is still unclear. In addition, there is mounting evidence to show that treating periodontal infections in a poorly controlled diabetic patient can help to bring blood sugar levels under control [52]. The risk assessment for progression to the oral sequelae of diabetes, especially periodontitis, requires a detailed study of glycemic control, including the patient’s diet, HbA1c, and postprandial glucose values.

The level of glycemic control is the major determining risk factor, and several studies suggest that individuals who ignore their diabetes or have trouble controlling their serum glucose level have a 2–3 times increased chance of developing periodontitis [53].

Longitudinal studies also reveal that diabetic people are more likely to experience progression of periodontitis. Patients with periodontitis, for instance, lose greater periodontal tissue support when diabetes is present, according to cross-sectional epidemiologic research [54]. Successful periodontal treatment has been shown to lower the circulating levels of C-reactive protein (CRP) and tumor necrosis factor (TNF)-α in people with diabetes, providing further evidence of its active role in inflammation [55].

Elevated serum C-reactive protein (CRP) levels were associated with a fourfold increase in risk for future diabetes, according to data collected in women’s health research [56]. Furthermore, CRP and the pro-inflammatory cytokines intefleukin-6 (IL-6) and TNF-α were linked to insulin sensitivity and the characteristics of insulin resistance syndrome in cross-sectional investigations [57].

Animals exposed to TNF-α for extended periods of time developed insulin resistance; conversely, TNF-α neutralization improved insulin sensitivity [58]. There exist multiple direct and indirect pathways through which TNF-α can bring about insulin resistance [59]. The expression of various adipocyte genes known to impact insulin sensitivity/resistance has been shown to be controlled by TNF-α, according to recent studies [60].

In individuals with systemically healthy periodontitis, it is unclear whether successful periodontal treatment significantly decreases the circulating levels of inflammatory markers like IL-6 or TNF-α [61]. In addition, patients with severe periodontitis have been shown to have higher CRP and IL-6 levels, which have been shown to decrease after periodontal therapy [62].

Periodontal disease and diabetes mellitus are examples of comorbid conditions. Despite their unique causes and effects, many environmental and genetic factors are shared by these disorders. Different people have different reactions to the same stresses in the environment, and these differences can be traced back to their unique genetic make-up. The role of genetics in the development of, and susceptibility to, these diseases is now well-established. Polymorphisms and genetic mutations interact synergistically with different environmental agents to cause susceptibility to, and determine the severity of, disease [63].

### 3.3. Dental Caries

Dental caries and DM have a tangled connection. There are inconsistencies in the research on the links between diabetes and tooth decay [64]. While Jones stated that DM increased the risk of caries [65], there was no statistically significant difference in the mean amount of caries between diabetic and non-diabetic patients in a study conducted by Arrieta-Blanco et al. [66]. Carious lesions were more common in those with diabetes (7.39%) than in people without diabetes (6.91%). Dental caries were more common among non-diabetics (32.3% vs. 13.6%) in a separate study of 600 patients (300 with diabetes and 300 healthy) [66].

Patients with diabetes may have fewer cavities because their diets contain more protein and fewer fermentable carbohydrates, according to research conducted by Bharateesh et al. [67]. Table 1 shows that diabetic individuals had a higher treatment demand compared to healthy subjects, but a lower rate of tooth decay.

A second study with comparable methods failed to show an increased risk of cavities in patients with type 1 diabetes compared to a control group of healthy people [68]. Hegde et al. [69] discovered that diabetic caries-active participants had significantly lower levels of salivary calcium and significantly higher levels of alkaline phosphatase.

Ageing, a high plaque score, and a poor unstimulated salivary flow rate were found to be substantially related to high caries incidence among diabetics by Siudikiene et al. [70].

Twetman et al. found that people with poorly managed diabetes had three times the rate of caries development compared to those who had improved glucose control, as we can notice in Table 2 [71].

The increased frequency of dental caries in patients with type 1 diabetes may be due to a combination of hereditary factors, oral cariogenic bacteria, diet, and oral hygiene, with this being a multifactorial disease.

Caries prevention in diabetic patients can be improved with additional research into the relationship between diabetic management and other factors of the diabetes phenotype and dental caries [72,73].

Additional research is necessary to explore the correlation between diabetes and dental caries, as well as tooth loss. If the existence of negative impacts of diabetes on dental caries and tooth loss is confirmed, the findings from this research would contribute to the development of intervention studies aimed at preventing or mitigating the occurrence of dental caries and tooth loss in individuals with diabetes. Furthermore, these findings have the potential to impact current clinical practice protocols and facilitate the development of novel public policies pertaining to diabetes.

## 4. An Update on Glycemic Management

In order to prevent systemic and oral problems, it is essential to maintain stable blood glucose levels, often known as glycemic control. This is why a management strategy is required. An interdisciplinary group consisting of the patient, his or her family, the attending physician, the family dentist, the dental hygienist, and the dietitian should work together to create this plan.

Several factors related to the patient should be taken into account when formulating the plan, including, but not limited to, age, school or work schedule and conditions, physical activity, medications (insulin or oral hypoglycemic agents), dietary habits, social situation, personality, cultural factors, the presence of complications (systemic and/or oral), and any other medical conditions [74].

Oral issues are one of many problems that have been linked to both type 1 and type 2 diabetes and high blood sugar. These issues generally emerge when the levels of blood glucose are not regulated. So, patients with uncontrolled DM experience a higher number of teeth affected at the coronal and pulp levels [75]. On the other hand, there is a twofold impact effect on dental health caused by hyperglycemia. First, an increase in the amounts of glucose in the saliva and gingival crevicular fluid facilitates the development of harmful microorganisms [76].

**Figure 3:**
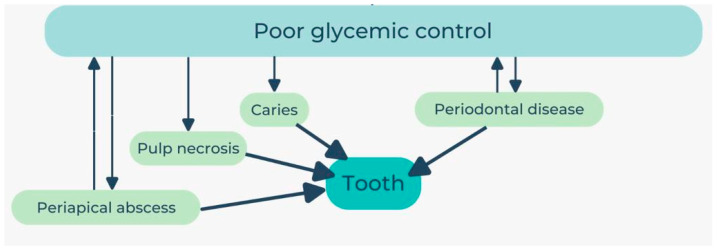
Connection between glycemic control and oral health.

In addition, it increases the exposure of oral tissues to aldose sugars, which stimulate nonenzymatic glycation and oxidation, and therefore, the development of advanced glycation end-products (AGEs) [77]. A concise scheme of these processes is represented in Figure 3 below:

Therefore, the regulation of blood glucose levels is a crucial aspect of managing diabetes in order to reduce the occurrence of related problems. Glycosylated hemoglobin (HbA1c) testing is commonly used to evaluate long-term glucose management (three to four months). Achieving glycemic control, as measured by percentage decreases in HbA1c, has been linked to lower risks of microvascular illness in prospective randomized clinical studies. In addition, epidemiological research supports evidence of strict glycemic control’s potential to lessen the prevalence of macrovascular disease [78,79,80,81].

The goal of medical care is to maintain a level of HbA1c of less than 7%, or an average blood glucose level of less than 150 mg/dL, on a three- to six-monthly basis; if the result is greater than 8%, treatment is indicated [82]. However, it has been established that factors beyond HbA1c’s reflection of glucose management may contribute to or modify the risk of problems [83].

Several international guidelines were produced to guide practice despite a lack of high-quality evidence on the inpatient management of diabetes. For the majority of critically ill patients with hyperglycemia, the American Diabetes Association (ADA) and the American Association of Clinical Endocrinologists (AACE) task force recommend aiming for a glucose concentration of 7.8–10.0 mmol/L (140–180 mg/dL), with a lower glucose target range of 6.1–7.8 mmol/L (110–140 mg/dL) for selected patients in intensive care units [84].

Pre-meal glucose concentration targets of fewer than 7.8 mmol/L (140 mg/dL) and random blood glucose concentration objectives of less than 10.0 mmol/L (180 mg/dL) are advised for non-critically sick individuals with hyperglycemia in the Endocrine Society guidelines and the ADA-AACE consensus statement [84].

New clinical evidence suggests that the magnitude of postprandial (after meals) hyperglycemic rises in blood glucose may be even more predictive of the likelihood and severity of problems [85,86]. Increased free radical production after a meal is linked to tissue toxicity and damage and, in the long run, may be linked to kidney failure in those with acute hyperglycemia. Independent of fasting blood glucose, there is an elevated risk of death in those who experience acute hyperglycemic spikes (or excursions) within the first two hours following a meal.

Controlling postprandial hyperglycemia should be at the center of all nutritional therapies for type 2 diabetes since it is a significant pathophysiological condition that contributes to the development and progression of micro- and macrovascular problems in the disease [87].

Carbohydrates are the primary factor in the postprandial glycemic response, making both their quality and quantity crucial factors in determining postprandial glucose levels. The amount of soluble fiber [88] and the Glycemic Index (GI) of meals [89] are therefore also important contributing factors to postprandial glucose responses, in addition to the amount and type of carbs consumed. Therefore, a low-GI/GL (glycemic load) diet as a whole may be insufficient when trying to manage postprandial glucose variations because it does not take into account the carbohydrate type and amount of each meal. It is crucial to pay attention to the nutrient composition of each meal independently in order to reduce hyperglycemic spikes in the postprandial state throughout the day [90]. Blood glucose levels, which serve as fuel for the body’s tissues, must be kept within a narrow physiological range (70–80 mg/dL when fasting and 140–160 mg/dL postprandial) at all times [90].

From 180 to 260 mg/dL, postprandial glucose levels increase the risk of microvascular illness [91]. Therefore, the focus of modern medical therapy has shifted to include the regular self-monitoring of blood glucose with a glucometer four to six times a day, usually before and after meals, to control one’s postprandial levels and prevent acute hyperglycemia and acute tissue toxicity [92].

Since tissues must retain and utilize the glucose that was consumed, the postprandial state places a substantial demand on metabolism. This is accomplished via the coordinated actions of the digestive system, the nervous system, the hormones in the gut, the secretion and action of insulin and glucagon, and the removal of glucose from the body following eating [93].

Engaging in a delicate balancing act of strict metabolic regulation entails potential hazards. Individuals with diabetes, specifically those with type 1 diabetes who administer multiple daily insulin injections or utilize an insulin pump [94], are susceptible to experiencing severe hypoglycemia or abnormally low blood sugar levels (commonly referred to as insulin shock). Such episodes can escalate to the point of life-threatening diabetic ketoacidosis (DKA) [94]. Diabetic ketoacidosis (DKA) and hyperglycemic hyperosmolar state (HHS) are the most serious and life-threatening hyperglycemic emergencies in patients with diabetes. In both conditions, insulin resistance and extreme hyperglycemia are hallmarks. Aggressive rehydration, insulin therapy, electrolyte replacement, and identification and management of precipitating factors are cornerstones of care for both DKA and HHS [95].

## 5. General Management Considerations

Patients who have not been diagnosed with dental conditions but exhibit the primary signs and symptoms of diabetes, such as excessive thirst, frequent urination, increased appetite, weight loss, and weakness, or display oral manifestations like dry mouth or candidiasis, should be directed to a physician for proper diagnosis and treatment.

It is imperative to identify patients who have been previously diagnosed with diabetes and, subsequently, to create a thorough medical intervention plan for such individuals, encompassing their pharmaceutical regimen, dietary plan, and glycemic management, while also addressing any systemic complications arising from diabetes.

In accordance with the guidelines established by the American Diabetes Association, it is crucial for health care professionals responsible for diabetes care to consistently deliver instructions and conduct regular evaluations pertaining to the appropriate methodology, interpretation of findings, and application of data acquired through the self-monitoring of blood glucose. This is essential for patients to make the requisite adjustments to their therapy [96].

The American Diabetes Association (ADA) revised its guidelines for treating diabetes in 2015, basing its decisions on a thorough analysis of the available evidence. This review prompted the ADA to revise its diagnostic criteria for type 2 diabetes and pre-diabetes [96]. Particularly noteworthy is the increased emphasis on diabetic patients’ dental health in the ADA’s 2016 recommendations. Comprehensive diabetic medical examination should include the “presence of common comorbidities including dental disease; recommendations for preventative care services including dental referrals,” as part of the construction of medical history [96].

Nevertheless, there is no correlation between an awareness of one’s heightened susceptibility to oral disorders, such as periodontal disease and xerostomia, or the receipt of guidance from diabetic care providers regarding the significance of frequent dental check-ups, and an increase in the utilization of dental services [97]. In a similar vein, it has been observed that the provision of guidance regarding the significance of maintaining glycemic control through dental care providers does not serve as a predictor for consistent glucose self-monitoring or adherence to annual visits with the diabetes care provider [98].

In the context of surgical interventions, it is imperative for a dentist to engage in consultation with the primary care physician of a patient diagnosed with diabetes mellitus. This collaborative discussion aims to address and accommodate any required modifications to the dental treatment plan, taking into consideration the patient’s systemic difficulties resulting from the aforementioned medical condition.

Diabetic patients undergoing periodontal or oral surgery, other than simple extractions, should be provided with postoperative dietary guidelines formulated in collaboration with the patient’s primary care physician and registered dietitian. Maintaining good glycemic control within diabetes requires a diet that is consistent in terms of overall caloric content and the protein–carbohydrate–fat ratio. It is advisable that patients receive guidance from their physician regarding dietary advice and potential adjustments to their medication dosages during the postoperative period following dental treatment [99].

Short morning appointments are preferable for reducing patients’ stress levels. Endogenous epinephrine production in a reaction to stress can counter-regulate insulin function, leading to increased glycogen breakdown in muscle (and, to a lesser extent, the liver) and a subsequent rise in blood glucose levels. Adults with diabetes who have no history of hypertension, or whose hypertension is effectively controlled, can receive epinephrine within the usual dosage range [100].

Individuals who have not yet been diagnosed with DM require the active participation of a dentist in order to facilitate early identification, assessment, and management. Patients with diabetes should see a dentist at least once every three months [101]. Before any dental work can be carried out, a thorough examination of the oral cavity and patient history is required [102]. The dentist should know how to treat undiagnosed or uncontrolled diabetes medically and be able to spot its symptoms.

Ultimately, the dentist has a significant role to play in helping patients to change their harmful health habits, especially those that contribute to the development of comorbidities. Epidemiologic, case–control, and cohort studies, for instance, provide substantial evidence for a causal relationship between cigarette smoking and diseases like diabetes and oral cancer [103,104]. The reported dangers appear to be at least equivalent to those found in the general population, as much of the research documenting the impact of smoking on health did not separately examine the results obtained for subsets of individuals with diabetes.

## 6. Diabetic Oral Complication Management

Systemic disorders, such as diabetes mellitus (DM), may be accompanied by abnormal oral signs and/or may stimulate the development and progression of oral diseases, and there is a close association between oral health and systemic health [105,106]. However, periodontal disorders (gingivitis and periodontitis) are a symptom of a larger problem and can affect the development and progression of systemic diseases including diabetes and heart disease [107,108]. Patients with certain diagnoses are considered “at-risk”.

The research into diabetics’ oral health knowledge is abundant. Studies have included knowledge items used to determine how well patients understood the risk of oral health problems due to diabetes, the importance of good diabetic control, and the preventive oral health behaviors (brushing, flossing, and regular dental visits) that could lessen that risk. Several factors have been hypothesized to play a role in the relationship between oral and systemic health, including genetics (gene polymorphisms), lifestyle (stress, habits like smoking, and high-fat diets/the consumption of highly processed foods), medication, microbial imbalances and bacteremia, and altered host immunity. Therefore, these factors serve as a catalyst for the development of periodontal disorders and other general diseases in a population that is already predisposed to them. More than half of diabetics are not aware of their elevated risk for periodontal disease and other oral health concerns, according to a large body of research [109,110].

Due to the negative effects of periodontal disease on diabetes and the positive effects of good oral health practices in reducing the risk of periodontal disease, it is crucial that people with diabetes receive risk assessments and dental referrals as part of their routine diabetes care [111,112,113]. Although numerous researchers throughout the world have evaluated diabetics’ understanding, beliefs, and behaviors in this area, no comprehensive analysis has been performed. Appropriate oral health knowledge or literacy is positively associated with excellent oral health behaviors like an increased frequency of brushing and dental visits [111] and good periodontal health [112]; hence, it is important to conduct such reviews. In addition, the social determinants of health [114] have an impact on people’s oral health behaviors, making it more likely that those from disadvantaged or lower socioeconomic backgrounds will experience the burden of oral disease due to their unhealthy habits, lack of knowledge and attitudes towards oral health, and reduced use of dental services.

An American study found that people who knew how to better take care of their teeth and gums were more likely to brush their teeth twice a day, floss once a day, and see a dentist twice a year (*p* = 0.01) [113]. Higher levels of schooling (*p* = 0.05) [115] and exposure to oral health education (*p* = 0.008) [113,116] were also significantly associated with proficient oral health knowledge. According to reports in the scientific literature, general practitioners and diabetes care providers did not give patients any information about oral health in the vast majority of cases [117,118,119].

Oral health care providers’ participation in DM risk identification strategies will improve screening and preventative measures against this illness. Dentists who are well-versed in the dental effects and dangers of DM can better help their patients [120,121]. Patients with diabetes should be encouraged to attend dentist appointments through diabetic and dental care facilities so that they can receive support and teaching in oral health knowledge. Patients with diabetes have an increased need for dental and medical management due to the correlation between systemic and oral health. Patients, doctors, and dentists should work together to improve the overall and dental health of diabetic patients.

Dentists should take part in group-wide educational programs to increase diabetics’ understanding of the need to protect their oral health. Dental professionals and government agencies both have a stake in preventing potentially serious issues through public education initiatives.

All diabetes care providers should make sure that their patients have an oral health review and referrals to a dentist, as is recommended by both international (International Diabetes Federation-IDF) and national (Royal Australian College of General Practitioners-RACGP) consensus recommendations [122,123].

Despite the correlation between diabetes and poor dental hygiene and the existence of clear guidelines, investigations conducted in different nations have shown that individuals suffering from diabetes have a notably decreased level of oral health understanding, consciousness, and compliance with good oral health behaviors [124].

The promotion and improvement of oral health should be a standard operating procedure for healthcare policymakers and doctors, according to an editorial published in The Lancet [125]. In this editorial, it was argued that ‘oral health is a neglected area of global health’. Policymakers and governments have, unfortunately, tended to perceive oral disorders as less significant than more life-threatening diseases, despite the fact that poor dental health predominantly affects morbidity rather than mortality.

Diabetes educators and other non-dental professionals should do more to improve diabetic patients’ oral health. This function has been investigated further in two investigations, one in Finland and one in Thailand [126,127]. Positive patient outcomes were observed when diabetes educators were instructed in preventative oral health measures (oral health education, instructions, and referral for dental visits) in both settings. Patients with diabetes who participated in these programs had lower levels of plaque and HbA1c, better oral health knowledge, attitudes, and practices, and an increased frequency of dental checkups [126,127].

Another key issue to consider when involving a non-dental professional in oral health promotion is the availability of proper oral evaluation tools The role of diabetes care providers in promoting oral health is increasingly highlighted in recent guidelines and studies, which is encouraging given the widespread neglect of oral health care in normal diabetes care.

Complications associated with diabetes mellitus (DM) can be avoided through lifestyle changes such as greater physical activity, improved nutrition, weight loss, and the control of blood pressure, cholesterol, and mental health disorders. Normal lifestyle adjustments, a healthy diet, regular exercise, and the use of antidiabetic medicines can help to prevent diabetes in high-risk persons who already have impaired glucose tolerance or fasting glucose [128].

Controlling blood glucose, learning about and practicing self-care for DM, and maintaining good dental health are all preventative approaches that can help to lessen the oral symptoms of diabetes. Health care costs for diabetics can be reduced by taking these steps.

## 7. Oral Health and Oral Management of Elderly Patients with Diabetes

The percentage of older patients who suffer from hyperglycemia or diabetes is unknown. The estimated prevalence of diabetes in persons aged 65–75 and >80 years old is 20% and 40%, respectively [129,130], according to cross-sectional research.

Poor oral health knowledge, beliefs, and behaviors are common in diabetics over the age of 65, as is ignorance about the link between diabetes and periodontitis [130].

Age at diabetes development is a differentiating factor among the elderly. Patients with diabetes over longer periods of time tend to have poorer glycemic control and a higher prevalence of microvascular complications [131]. In addition to managing blood sugar, a greater emphasis on oral health education is needed to help diabetic elderly individuals with their oral health issues.

Evidence suggests that patients can help each other strengthen their beliefs, improve their unhealthy lifestyle patterns, and acquire positive oral behaviors through communication, preaching, and learning from each other’s experiences [132]. More than half of patients who visited a clinic for at least a year felt it was unnecessary to have an outpatient oral examination, according to a survey conducted outside of the United States, suggesting that a strong belief in oral health had not yet been created [133].

More than half of older people with diabetes, according to surveys by Li Yanling and colleagues [134], are uninformed about the importance of oral health. The reason for this is because education on diabetes and dental health is not widely provided, and when it is, it is often only at the level of traditional knowledge and lacks comprehensiveness. Li Yanling’s study [134] demonstrates that few people are aware of the intrinsic connection between the two. Patients only obtain some grasp of this connection after they have experienced oral health issues. Unfortunately, preventive dental healthcare is not a part of diabetes treatment programs, and more than half of patients have never received information regarding diabetes management and mouth health from their healthcare providers.

According to the findings of other research, there is a pressing need to include multiple methods and channels of oral health education for elderly patients with diabetes in order to decrease the prevalence of oral diseases [135].

Due to advances in medicine, the modern definition of health encompasses more than just the absence of illness; it also includes a robust emotional, mental, and social life [136]. Thus, it is more in line with contemporary views of health to include the “quality of life” evaluation measure into the field of stomatology in order to assess the effect of oral diseases on patients’ physical, psychological, and social capabilities. Patients with diabetes experience a decline in their oral quality of life over the course of the disease’s progression [137].

Physiological functions, behavioral impacts relating to oral health, cognitive difficulties, and psychological aspects of life are most affected by oral health issues [138], such as periodontal disease, in older individuals with diabetes. Patients’ oral health can be improved by health education that raises their consciousness about diabetes and its effects on their mouths, deepens their understanding of oral healthcare, alters their poor habits, and instills more positive ones [139].

### 7.1. Challenges in Preventive Dental Care of Older Adults with Diabetes

Increased counterregulatory hormones (cortisol, catecholamines, growth hormone, and glucagon) and proinflammatory cytokines interfere with carbohydrate metabolism, causing hyperglycemia in patients with acute medical or surgical conditions. This results in excessive hepatic glucose production and decreased glucose uptake in peripheral tissues.

When choosing glucose-lowering drugs for senior patients, healthcare providers must take into account the patients’ comorbidities, preexisting diabetic problems, and medication management to reduce the risk of adverse drug events and drug interactions.

Hyperglycemia and diabetes are more common in the elderly because of aging-related physiological changes [140,141]. Muscle and adipose tissue, in particular, become less sensitive to glucose and more resistant to insulin as we age [142]. Increasing insulin resistance in the elderly has been linked to abdominal obesity, elevated circulation levels of free fatty acids, and inflammatory markers such as tumor necrosis factors and interleukin 6 [143,144]. Drugs with negative effects on carbohydrate metabolism, such as diuretics, beta-blockers, and glucocorticoids, also contribute to the onset of hyperglycemia in the elderly.

Due to its link to increased morbidity and mortality, avoiding or minimizing hypoglycemia is particularly critical [145]. Ineffective glucose monitoring decreased counter regulation, a lower symptom threshold, and an increased the prevalence of hypoglycemia with age [146].

There are two pathways that contribute to the development of diabetes problems. To begin, glucose is processed via the polyol route into the tissue-damaging enzyme sorbitol byaldose reductase, among other diabetic problems. Second, the deposition of advanced glycosylation end products (AGE) in certain organs leads to structural and functional changes and a host of difficulties [147]. AGE is formed when glucose binds to proteins, lipids, and nucleic acids. Cells produce atheroma deposits, which damage the basal membrane and lumen and hence reduce the ability of the immune system to respond [134]. Due to impaired oxygen transport across the capillary wall, diabetic patients are more likely to develop infections, especially those caused by anaerobic bacteria.

Preventing cardiovascular and microvascular illness is an essential part of managing diabetes, and the early detection and care of complications are a crucial part of this process. In addition to medications used to lower glucose, blood pressure, and lipids, lifestyle interventions like education, self-management, and self-monitoring are crucial [148]. The management of diabetes is improved with structured education courses [149,150,151,152], and similar programs are being developed for periodontitis [153,154].

Healthcare practitioners have unique hurdles while caring for older patients with diabetes. There are two main sources of difficulty in this context: the increasing prevalence of diabetes and its complications, and the concomitant occurrence of geriatric syndromes.

Common geriatric comorbidities, such as frailty, fall risk, dementia, functional decline, and the necessity for placement in long-term care homes [150], should be taken into account in the management of older adults with diabetes. Numerous psychosocial issues, such as despair, poverty, and social isolation, further compound these difficulties [151,152].

Case managers, social workers, and whoever else is well-versed in local counselling options are an invaluable asset to any diabetes care team. Protective services, including medication compliance monitoring, food planning, and transportation, are all areas in which case managers shine [150].

The best way to control diabetes in the elderly is yet unknown, thus treatment must be tailored to the specific needs of each patient. The patient’s or caretaker’s preferences and goals for therapy, the existence of co-morbid conditions, the patient’s level of disability and frailty, and the patient’s predicted life expectancy are all factors that should be taken into account when developing an individualized treatment plan [153,154].

Even among medical professionals who are aware of the correlation between diabetes and oral health, a lack of knowledge and confidence on the subject has been cited as a major barrier to discussing dental health with patients with diabetes [155]. Doctors’ referrals of patients with diabetes to the dentist do not increase appreciably even when they are aware of the link between the two conditions’ oral health and overall health.

It has also been speculated, though never evaluated objectively, that dentists may be reluctant to refer patients for private care out of fear that their patients may be unable to afford it [156].

Improved communication and cooperation between stomatology, endocrinology, geriatrics, and other relevant fields is essential if diabetic seniors are to acquire the knowledge, beliefs, and behaviors necessary to improve their oral health and boost their chances of recovery [157].

The need to prioritize individual preferences and quality of life has led to significant shifts in diabetes management during the past two decades. While this has been put into practice in the community, it is well known that allowing for individuality in a hospital or other institutional setting can be fraught with difficulties [157,158].

Newer insulins, with their enhanced pharmacodynamic consistency and lower risk of hypoglycemia, should be administered first if insulin therapy is necessary. People who produce an adequate amount of their own insulin may benefit more from taking one of several oral medications that do not result in hypoglycemia when administered alone. It has been discovered that some of these oral medications have cardioprotective effects [159].

Managing other risk factors for cardiovascular diseases in people with diabetes would involve the use of antihypertensive medications, cholesterol-lowering medicines, and low-dose aspirin. As additional information becomes available, however, the optimal ranges for controlling blood pressure and LDL cholesterol continue to shift.

Strengthening treatment is preferable, but the medication schedule must be easy to follow, inexpensive, and secure to be effective. Generating realistic and attainable goals is essential since many seniors value autonomy preservation more than meeting glycemic targets. Rather than aiming to normalize blood glucose levels or meet predetermined blood pressure and LDL cholesterol targets, diabetes care in older persons with functional limitations and reduced life expectancy should focus on improving the quality of life of the individual [160].

An assessment of physical and cognitive function, as well as screening for geriatric syndromes, should be part of the treatment plan for elderly patients with diabetes. Then, when discussing the objectives and methods, double-check that you’ve thought of everything, especially whether or not the patient can actually carry them. Notably, untreated cognitive or functional impairments may explain why some elderly individuals do not respond to treatment. With the proliferation of technological tools, telemedicine approaches can also be investigated and evaluated for viability. Self-care measures developed in the twenty-first century will help the aging population by reducing the strain on healthcare systems and allowing for self-monitoring in the comfort of one’s own home, workplace, or other setting [20] Self-management approaches need to be incorporated into daily life in order for self-care of a chronic condition to be maintained [161].

Self-monitoring is a new phenomenon but it has already made significant strides towards becoming an integral part of people’s daily lives. More and more people are using apps on their mobile devices to track their health [162]. However, inadequate regulation in the technology sector allows unproven [163] self-monitoring gadgets to enter the market, potentially leading health consumers to make inappropriate changes to their self-care routines.

**Figure 4:**
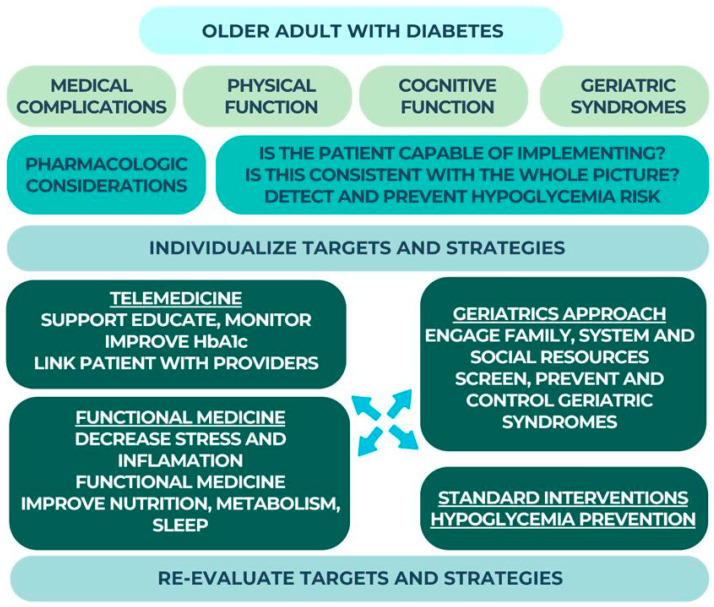
Treatment strategies for diabetic elderly patients.

Last but not least, supplementary measures to lessen inflammation and stress, enhance nutrition, and back up proven interventions, particularly for patients who may not be interested in or who do not react to conventional therapies. In Figure 4 below we summarized an integrated strategy to treating diabetes in the elderly.

### 7.2. Challenges in Preventive Dental Care of Young Patients with Diabetes

Children and teenagers are at high risk for developing diabetes. One in every 600 school-aged children has diabetes, and 0.02% of the youth population, or 186,300 children and adolescents under the age of 20, have diabetes [164].

A lack of research into the occurrence of periodontal disease in diabetic children younger than 6 years of age is concerning given the fact that diabetes can impact the periodontal tissues in children as early as the sixth year of life. The connection between diabetes and the onset of gingival or periodontal disease in young children is an area in which very little research has been conducted [165,166].

Other research has looked at how gingival inflammation relates to how well a child’s metabolism is controlled, and they have shown that children with diabetes who have poor glycemic control tend to have higher gingival index scores [167].

Their findings highlight the significance of stressing good oral hygiene in order to prevent future periodontal complications in diabetic patients, as children with diabetes have a significantly higher risk for gingival bleeding, and diabetes-related oral complications affect the primary periodontium as early as age 6, possibly earlier [167].

Children diagnosed with DM exhibit several oral health impairments, including alterations in saliva quantity and composition, dental caries, periodontal health, oral microbiota, dental growth, tooth eruption, and microbiological oral infections. These findings suggest that the oral health status of children with DM can be compromised. There is also a suggestion that certain elements stated before may exert a contrasting influence on the progression of diabetes [168]. 

## 8. Discussions and Future Perspectives

There is a strong association between diabetes mellitus and dental diseases. Diabetes affects individuals across many age groups, and its incidence has undeniably increased as a result of factors such as lifestyle modifications and extended life expectancy, among others.

Several groups, including those working in diabetes treatment, oral healthcare, and policy, stand to benefit from this review’s findings. Diabetes specialists need to do more to encourage their geriatric patients to take care of their teeth. Regular dental exams are strongly recommended, and dentists should inform, patients, especially those who are geriatric, of their increased risk of developing oral health concerns. In order to incorporate oral health promotion into their practice, diabetes care providers may also need to improve their own understanding in this area, as an interdisciplinary approach.

The mouth is one of the most vulnerable areas to the destructive effects of diabetes. Diabetic issues in the mouth are nearly unavoidable, although they can be mitigated by maintaining frequent doctor and dental appointments.

Poor glycemic control has been linked to an increased incidence of oral symptoms in diabetics, compared to those whose blood glucose levels are well-managed. Diabetics face unique challenges in maintaining stable blood sugar levels, making the prevention and management of oral problems, particularly periodontitis and gingivitis, of paramount importance.

Dentists play a significant role in the prevention and management of oral problems related to DM, and their contributions should never be overlooked or minimized.

Therefore, it is reasonable to include dental staff in geriatric patients’ diabetes care. Since many people see their dentist frequently (e.g., every 6 months, often more frequently than they see their medical practitioner), and because intra-oral findings may raise suspicions of undiagnosed diabetes, the dental team may represent an important screening resource for patients susceptible to global disease. When it comes to helping patients to change their habits, the dental team (especially dental hygienists) might be an underused means for medical professionals.

## 9. Conclusions

The prevention of diabetes and a better quality of life in geriatric patients can result from increasing their level of knowledge regarding this disease and its connection to oral health and other oral complications.

Professionals in the field of oral medicine should educate their patients, including geriatric ones, with diabetes on the need to maintain strict control of their condition so as to reduce the hazards to their oral health.

In order to improve access to dental treatment for this vulnerable segment of the population, policymakers must design and implement standardized oral health care standards and oral health promotional resources for diabetes care settings, as well as suitable referral mechanisms.

## Figures and Tables

**Table 1:** The Bharateesh [67] study outcomes.

Patients	Periodontal Disease	Dental Caries
With DM	92.6%	13.6%
Without DM	83%	32.3%

**Table 2:** Studies outcomes about caries and DM connections.

Authors	Study	Outcomes
Jones et al. [65]	This research compared the oral health status and habits of a sample of people with diabetes to those found in a national survey in the United Kingdom.	DM increased the risk of caries in the study group.
Arieta-Blanco et al. [66]	Study of the oral manifestations of diabetes and a comparison between the status of dental hygiene and the incidence of dental caries in a diabetic population and a control group.	Carious lesions were more common in those with diabetes (7.39%) than in people without diabetes (6.91%)
Bharateesh et al. [67]	A comparison of the rates of tooth decay and other common dental problems in diabetic and non-diabetic adults in southern India.	Patients with diabetes may have fewer cavities (13.6%) because of their diets comparing with DM patients (32.3%)
Miralles-Jorda et al. [68]	Patients with type 1 (insulin-dependent) diabetes mellitus (IDDM) were studied to determine the possibility that they experience oral issues as a result of the disease, or that a specific abnormality of the oral cavity might be viewed as pathognomonic of diabetes	Higher risk of cavities in patients with type 1 diabetes compared to healthy people
Hedge et al. [69]	Saliva alkaline phosphatase and calcium ion levels were compared and evaluated between caries-active type II diabetes mellitus patients and non-diabetic controls.	Salivary calcium levels were substantially lower in diabetic caries-active subjects, but alkaline phosphatase levels were significantly greater.
Siudikiene et al. [70]	The study compared the dental caries and salivary condition of children with and without type 1 diabetes mellitus.	High caries levels in diabetics were significantly associated with age, plaque score, and decreased unstimulated salivary flow rate, but were not associated with the level of metabolic control of diabetes.
Twetman et al. [71]	The study investigated the relationship between caries risk and glucosylated hemoglobin.	Patients with poorly managed diabetes had 3 times the rate of caries development compared to those who had improved glucose control.

## Data Availability

All data are available from the corresponding authors upon reasonable request.
